# Respiratory incidents in response to air quality deterioration in the summer and early autumn season

**DOI:** 10.1371/journal.pone.0335063

**Published:** 2025-10-22

**Authors:** Ewa Niewiadomska, Małgorzata Kowalska, Aleksandra Micek, Elwira Zajusz-Zubek

**Affiliations:** 1 Department of Epidemiology and Biostatistics, Faculty of Public Health in Bytom, Medical University of Silesia, Katowice, Poland; 2 Department of Epidemiology, Faculty of Medical Science, Medical University of Silesia, Katowice, Poland; 3 Student of Faculty of Energy and Environmental Engineering, Silesian University of Technology, Gliwice, Poland; 4 Department of Air Protection, Faculty of Energy and Environmental Engineering, Silesian University of Technology, Gliwice, Poland; University College of Medical Sciences, INDIA

## Abstract

The paper presents the results of an ecological study completed within the warm seasonal period of 01/07/2024–31/10/2024. The aim is to assess the relationship between ambient air quality deterioration and acute respiratory incidents occurring in the inhabitants of Gliwice (Poland; Upper Silesian agglomeration). The number of daily medical services (MS) due to respiratory diseases and illnesses was obtained from the register of the National Health Fund (Silesian Voivodeship Branch in Katowice). The daily mean values of aerosanitary factors in Gliwice came from the mobile air pollution laboratory of the Silesian University of Technology in Poland. The relative risk (*RR*) of MS was estimated using a Poisson log-linear model considering independent environmental factors (meteorological factors and air pollutant concentrations) or confounding factors (astronomical season and day of the week). The highest number of MS due to respiratory diseases refers to acute nasopharyngitis and asthma exacerbation, mostly in children aged 0–9 years. A significant impact was observed in the case of particulate matter concentration increase and number of MS due to acute nasopharyngitis, laryngitis and tracheitis, pneumonia, asthma exacerbation, in the case of nitrogen oxides appropriately acute nasopharyngitis, bronchitis and/or bronchiolitis, pneumonia, asthma exacerbation. Additionally, a longer time of exposure was associated with a higher risk of MS due to respiratory incidents. The increase in wind speed and relative humidity was significantly associated with a higher number of bronchitis and asthma exacerbation while a higher temperature and higher 8-hour ozone concentration remain protective of both MS. The results are essential for effectively communicating environmental health risks, educating the public about potential threats, and pressuring legislators responsible for legislation and risk management.

## Introduction

Ambient air pollution and its impact on the health of the inhabited population of Silesian Voivodeship (southern part of Poland) have been the subject of scientific research and public debate for many years [1–10]. Close cooperation between public health experts and institutions that assess environmental health risks causes a more aware society of the hazard, which is reflected in numerous protests and activities aimed at improving environmental quality [11,12].

Poland is located in a temperate climate zone, characterized by variable weather conditions and four distinct seasons. The urbanized and industrialized character of the Silesian Voivodeship is associated with serious ambient air pollution [1–10]. Seasonal conditions characterized by low temperatures and elevated air humidity create favorable circumstances for the development of winter smog [3–7]. In turn, high temperatures combined with high density of communication routes lead to increased levels of nitrogen oxides and ozone [8,9]. It should be emphasized that the majority of cited papers reports on the colder season of the year with lower air temperatures and higher daily concentrations of particulate matter. Observed climate changes with simultaneous increases in traffic emissions make it necessary to assess the relationship between ambient air quality and respiratory health problems, also during the warmer season (summer and early autumn). This is particularly significant for sensitive subpopulations, i.e., children and the elderly [13], and short-term effects related to ambient air pollution, including fine particulate matter. The most frequently mentioned are inflammatory reactions of the lungs and bronchi, acute respiratory symptoms such as cough, wheezing, and difficulty breathing, but also an increase in the daily number of hospitalizations and outpatient admissions, an increase in specific mortality, and an increase in medication use [14]. Moreover, it was demonstrated that a longer time of exposure led to a higher risk, with certain delays in health response over time (significant delays were observed for hospitalizations compared to outpatient visits). This observation is in line with other published data [8,15–17]; the effect depends on the internal dose, with the dose related to the time of exposure.

Although it is difficult to identify a dominant pathomechanism of interaction between particulate matter and respiratory or cardiovascular diseases, there seems to be a consensus that even a low increase in pollutant concentrations leads to many changes in the human body, ranging from the initiation of oxidative stress and local inflammatory processes in the respiratory system to rheological disorders, vasoconstriction, and consequently myocardial infarction and even stroke [18]. Previously published data indicate that even young and healthy adults engaging in physical activity are sensitive to the deteriorated air quality, which manifests in FeNO level increase (a marker of the inflammatory process) after an increase of PM_10_ and nitrogen dioxide NO_2_ concentrations [10].

These conclusions are based primarily on research conducted under conditions typical of the cold season. It seems that is important to recognize if ambient air quality measured in the warm season has an impact on the respiratory health of residents living in an urbanized, densely populated region of Poland with significant exceedance of limit value pollutants’ concentrations [19]. According to the authors’ knowledge, there are no such studies in this region of Europe.

The results obtained will support future awareness of health risks in society and the development of environmental health prophylaxis. Evidence plays a crucial role in effectively communicating environmental health risks, educating the public about potential threats, and applying pressure on politicians responsible for legislation and risk management. Collecting data on the cause-and-effect relationships between common hazards in the studied region and their actual health impacts helps to bridge the knowledge gap and raise awareness about necessary environmental changes.

### The aim

The study aimed to recognize the relationship between daily number of medical services due to acute respiratory incidents (J00-J99 according to ICD-10) and ambient air quality deterioration during the warm season (01/07/2024–31/10/2024) in the population of Gliwice city.

## Materials and methods

The study was an ecological study that used secondary epidemiological data from the registry of the Silesian Voivodeship Branch of the National Health Fund in Katowice (NFZ). The work is not a medical experiment therefore the approval of the Bioethics Committee was not required.

Data on medical services (MS) included only residents of Gliwice city and concerning the following causes (codes according to ICD-10): J00 – acute nasopharyngitis (cold), J04 – acute laryngitis and tracheitis, J10 and J11 – influenza, J12–J18 – pneumonia, J20–J21 – acute bronchitis and/or bronchiolitis, J45–J46 – asthma exacerbation or status asthmaticus. The database included dates (during the study period from 01/07/2024–31/10/2024) and the number of medical services (MS) per day. Additionally, a tabular distribution of all MS cases was obtained by patient age (from 0 to 111 years) and type of service (outpatient, inpatient). Such selection was based on one’s own experience and bibliography [14,20–25]. The registry data were limited to Gliwice, a city located in the Silesian Voivodeship, with a population of 169,915 (as of 2023) and an area of 134.2 km^2^ [26]. The city of Gliwice is part of the most urbanized and industrialized agglomeration in Poland and Central and Eastern Europe – the Upper Silesian and Zagłębie Metropolis, with the largest interchange in this part of Europe.

According to the seasonal classification relevant to southern Poland’s temperate climate, “summer” encompasses July and August, and “early autumn” refers to September and October. During July–October, Gliwice exhibits a mixed emission profile driven by industrial activities, intensive road traffic, and residential solid-fuel combustion (coal and wood) within the city and neighbouring municipalities. While average levels of PM₂.₅, PM₁₀, and NO₂ are generally lower than in winter, short-term increases can occur during stagnant atmospheric conditions (low wind speeds and weak boundary-layer mixing) and near major roads. Regional photochemistry may elevate O₃ during this period, whereas at traffic-dominated sites within the city, the near-road reaction between nitric oxide (NO) and ozone (O₃), often termed ozone scavenging, depresses local O₃ relative to urban-background or peri-urban locations.

Aerosanitary conditions of ambient air (concentration of PM_2.5_ as well as SO_2_, NO, NO_2_, NO_x_, O_3_, CO, and CO_2_) were obtained from measurements of mobile air pollution laboratory located on the campus of the Silesian University of Technology in Poland (Poland, Gliwice, Konarskiego 20B, 50.292934N, 18.682164E). PM_10_ concentrations were gathered from a regional ambient air quality monitoring station in Gliwice of GIOŚ (Chief Inspectorate of Environmental Protection, Poland, located in Gliwice, Mewy 34, 50.279481N, 18.655736E). Hourly measurements of each aerosanitary factor (pollutant concentrations, temperature, relative humidity, atmospheric pressure, and wind speed) were averaged to daily mean values (from 0:00–23:00 each day). For ozone (O_3_) and carbon monoxide (CO), the maximum measurements of 8-hour averages (O_3_-8h, CO-8h) were determined.

Both sites are within the contiguous built-up area of Gliwice and are away from immediate kerbside influence. The campus mobile laboratory operated in a mixed academic and residential setting and was treated as an urban-background location. The air-quality monitoring station in Gliwice, operated by the Chief Inspectorate of Environmental Protection (GIOŚ) as part of the national monitoring network, is classified as urban background. Using the population and area values already reported in the manuscript (169,915 in 2023 and 134.2 km^2^), the citywide density is approximately 1,266 inhabitants per km^2^. Taken together, these placements are intended to provide city-level exposure proxies that reflect area-wide urban conditions rather than measurements dominated by a single emission source.

Finally, we assessed the risk of daily number of medical services (both outpatient visits and hospitalizations) due to acute respiratory incidents which were related to hazard increase.

### Ethics statement

The study was based on anonymized secondary health data obtained from the National Health Fund (NFZ) branch in Katowice. Permission to use the data was granted by the NFZ for academic research. According to national regulations and institutional guidelines, the use of anonymized, aggregated data for non-interventional research does not require approval from a bioethics committee.

The NFZ authorised use of de-identified, aggregate health-service data for research; no IRB/REC review and no informed consent were required for anonymised, non-interventional analyses, in line with national regulations and GDPR.

### Statistical analysis

Statistical data analysis was conducted using descriptive and analytical statistics. Exposure to ambient air pollutants and meteorological factors was presented as a value of arithmetic mean (*X*) and standard deviation (*SD*), as well as a median (*Me*) and interquartile range (*IQR*). The relationship between respiratory health and exposure was assessed by multivariate analysis with the Poisson log-linear method. Confounding variables such as astronomical season (autumn vs. summer) and day of the week (workday vs. non-workday) were controlled in the model. In the study period, no influenza or COVID-19 epidemics were observed. A model function verified in our previous studies [14] was adopted:


$$log\left(E\left(y_{t}\right)\right)=\varepsilon_{t}+\sum_{i=\text{1}}^{n}\beta_{i}{p_{i,l}(x}_{t})$$ (1)

Where *E(y*_*t*_*)* is the expected number of MS (dependent variable), *p*_*l*_*(x*_*t*_) represents independent variables (environmental or confounding factor) considering the lag effect of the MS for period *l*, and *β* denotes the regression coefficient. Only the se*l*ected parameter and confounding variables, such as astronomical season and day of the week, were considered in the multivariate models, due to significant, strong interdependencies of meteorological factors and air pollutant concentrations (assessed using Spearman’s correlation coefficients and their statistical significance).

Changes in the number of MS in response to an increase in pollutant concentration by one unit (by the value of *IQR* or by 10 μg/m^3^ or 1 unit) were estimated. The exposure was presented in two variants. The first consideration was on health delays (*l* = 1, 2, 3,..., 10 days related to the measured concentration on a given day) in response to pollutant concentrations – the number of MS for the *i*-th day was combined with the exposure level for the *i-l* day. The second variant took into account the moving average concentrations of pollutants from 1, 2, 3,..., 10 days preceding the MS – the number of MS for the *i*-th day was combined with the arithmetic mean of the *l* previous days. Such an approach to exposure assessment is frequently utilized in studies of environmental epidemiology [27,28].

The results of the multivariate analysis were presented as a relative risk (*RR*) of MS due to an increase in exposure by one unit *Δ*x = const**. The following formula was used to estimate *RR*:


$${RR}_{i}=e^{{const\cdot \beta }_{i}}$$ (2)

Where *β*_*i*_ denotes the regression coefficient, while *const* represents the unit (here: *IQR* or 10 μg/m^3^, or 1 unit). The maximum values of risk ratio *RR*_*max*_ were interpreted in the description of the results. The chosen model of regression is in line with the well-known and previously described methods used in ecological types of studies [29–31].

The criterion of statistical significance was set as *α < 0.05*. To create the database and for descriptive analysis, the available formulas of MS Excel 2013 (MS Office Poland) were used. All computations were performed using Statistica version 13 software [TIBCO Software Inc. 2017, Statistica (data analysis software system), version 13] and R version 4.1.1 software [2021 The R Foundation for Statistical Computing].

## Results

### Pollutants concentrations and meteorological conditions

Table 1 represents aerosanitary data (pollutants concentrations and meteorological conditions) measured in the study period (i.e., from 01/07/2024–31/10/2024), as well as in periods corresponding to calendar seasons, summer (01/07/2024-22/09/2024) and autumn (23/09/2024–31/10/2024), respectively.

**Table 1:** Average values of particular air pollution concentrations and meteorological parameters in Gliwice city, separately in the whole studied period and particular season of the year.

Particular risk factors (pollutants and meteorological conditions)	01/07/2024-31/10/2024 TOTAL		01/07/2024-22/09/2024 SUMMER		23/09/2024-31/10/2024 AUTUMN	
	X ± SD	Me (IQR)	X ± SD	Me (IQR)	X ± SD	Me (IQR)
PM_10_ [µg/m^3^]	23.4 ± 12.3	21 (17.5)	22.5 ± 12	20.2 (15.7)	25.5 ± 12.8	23.4 (17.2)
PM_2.5_ [µg/m^3^]	17.4 ± 7.8	15.8 (9.6)	16 ± 6.3	14.9 (7.7)	20.2 ± 9.6	18.9 (10.4)
SO_2_ [µg/m^3^]	12.5 ± 8.9	18.7 (17.7)	17.1 ± 7	20 (2.5)	2.9 ± 2.2	2.4 (2.4)
NO [µg/m^3^]	10.7 ± 9.1	7.6 (9.9)	7.6 ± 6.8	5.2 (6.8)	13.8 ± 10.2	9.9 (13)
NO_2_ [µg/m^3^]	23 ± 9.9	21.6 (12.5)	28.1 ± 10.1	26 (13.1)	17.7 ± 6.3	17.5 (8.2)
NO_X_ [µg/m^3^]	39.3 ± 19.3	34.7 (25.4)	39.7 ± 18.4	35.7 (22.9)	38.8 ± 20.4	34.7 (27.8)
O_3_ [µg/m^3^]	55.8 ± 18.5	57.5 (24.5)	63.1 ± 14.8	63.3 (20.4)	38 ± 14.1	33.3 (22.9)
O_3_-8h [µg/m^3^]	90.3 ± 30	86.5 (48.4)	102.4 ± 24.8	105.2 (35.9)	60.6 ± 19.1	62 (21.4)
CO [mg/m^3^]	0.3 ± 0.2	0.3 (0.2)	0.3 ± 0.1	0.3 (0.1)	0.5 ± 0.2	0.4 (0.3)
CO-8h [mg/m^3^]	0.5 ± 0.3	0.4 (0.2)	0.4 ± 0.1	0.4 (0.1)	0.7 ± 0.3	0.6 (0.4)
TEMPERATURE [°C]	18.2 ± 5.4	19.2 (9.1)	21.2 ± 3.4	21.3 (5.1)	11.9 ± 2.8	11.1 (4.3)
HUMIDITY [%]	70.5 ± 11.3	71.2 (14.7)	67.3 ± 11.4	66.6 (16.1)	77.3 ± 7.7	77 (11.6)
PRESSURE [Hpasc]	991.5 ± 6.2	990.6 (7.9)	990.7 ± 4.5	990.3 (5.8)	993.2 ± 8.7	995.5 (13.6)
WIND SPEED [m/s]	1.1 ± 0.4	1 (0.5)	1.1 ± 0.4	1.1 (0.5)	0.9 ± 0.3	0.9 (0.4)

*X*, mean value; *SD*, standard deviation; *Me*, median; *IQR*, interquartile range.

**Fig 1:**
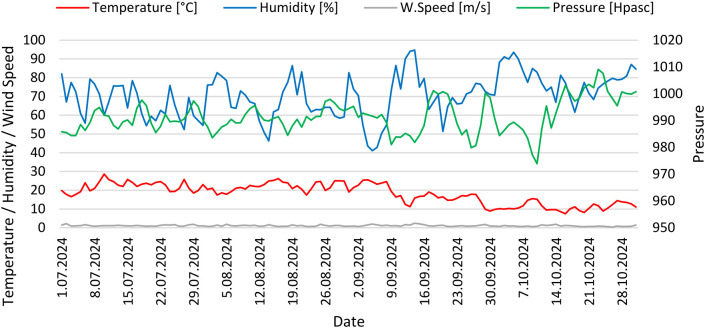
Daily mean value of meteorological parameters registered in Gliwice, in the whole study period (01/07/2024-31/10/2024).

The summer period, especially July and August, was characterized by high and relatively stable ambient air temperatures ranging from 16.6°C to 28.6°C, frequent relative humidity drops, and air stillness (Fig 1). The meteorological situation became more dynamic at the beginning of September, daily average temperatures dropped, and there was observed variability in relative humidity and atmospheric pressure.

### Epidemiological situation – medical services (MS) due to acute respiratory diseases

During the study period, a total number of 49,085 medical services (MS) due to acute respiratory diseases (J00-J99 according to ICD-10) were registered in Gliwice. Most of them were outpatient visits (47,721; 97.2%), while the remaining services were hospitalizations (1,373; 2.8%). Mostly, the group of beneficiaries included patients aged 0–9 years (S1 Table). S2 Table shows both, the number and percentage of MS according to particular diseases defined by ICD-10 classification. The majority of outpatient visits and hospitalizations referred to acute nasopharyngitis (J00; n = 8,329; 17.0% of the total) and asthma exacerbation or status asthmaticus (J45-J46; n = 4,917; 10.0% of the total). Other MS included acute bronchitis and/or bronchiolitis (J20-J21; n = 2,313; 4.7% of the total), acute laryngitis and tracheitis (J04; n = 1,824; 3.7% of the total), pneumonia (J12-J18; n = 1,661; 3.4% of the total), and influenza (J10 and J11; n = 237; 0.5% of the total).

**Fig 2:**
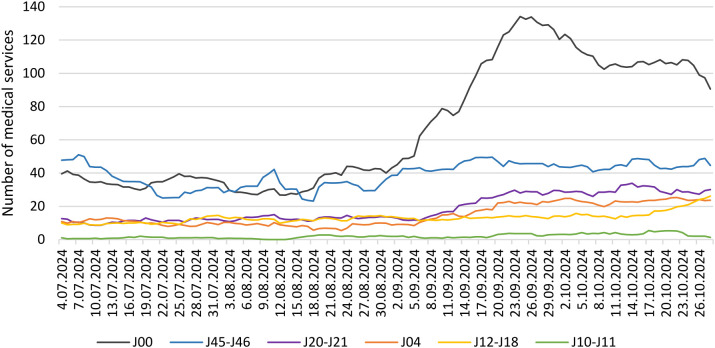
Number of medical services (MS) provided due to particular acute respiratory diseases in Gliwice, in the whole study period (01/07/2024-31/10/2024).

It should be noticed that the number of particular medical services due to respiratory diseases varied in time, with more cases recorded in the early autumn period (Fig 2). At the beginning of September, air temperatures were lower, and the start of the school year caused a noticeable increase in the number of cases of acute nasopharyngitis (cold) J00, as well as acute bronchitis and/or bronchiolitis J20-J21 (Table 2). For colds, children aged 0–9 years were the most frequently affected (43.4% in total), while for asthma, the highest percentage of illnesses occurred in both groups, children aged 5–9 years (12.9%) and adults aged 65–69 years (10.8%) (S2 Table).

**Table 2:** Average number of medical services (MS) provided due to respiratory diseases in the whole study period and separately in both seasons of the year.

Particular respiratory problems registered in Gliwice	Total 01/07/2024-31/10/2024		Summer 01/07/2024-22/09/2024		Autumn 23/09/2024-31/10/2024	
	X ± SD	Me (IQR)	X ± SD	Me (IQR)	X ± SD	Me (IQR)
J00-J99	399.1 + 260.7	429 (65-630)	324.3 + 200.5	392 (52.5-460)	560.2 + 302.4	696 (71-762)
J00	67.7 + 56.8	49 (21-122)	45.8 + 36.3	39.5 (15-59.5)	114.9 + 64.3	139 (30-159)
J04	14.8 + 11.3	13 (5-22)	10.6 + 7.4	11 (4-15.5)	23.9 + 12.8	27 (15-31)
J10-J11	1.9 + 2.5	1 (0-3)	1.3 + 1.8	0 (0-2)	3.3 + 3.2	2 (1-5)
J12-J18	13.5 + 8.9	14 (5-19)	11.7 + 6.9	12 (5-17.5)	17.4 + 11.4	17 (6-25)
J20-J21	18.8 + 14.6	17 (6-27)	13.5 + 9.6	14 (4-19.5)	30.2 + 17	33 (12-40)
J45-J46	40 + 28.2	47 (2-62)	36.9 + 27.2	42.5 (1-57.5)	46.6 + 29.4	54 (3-69)

*X*, mean value; *SD*, standard deviation; *Me*, median; *IQR*, interquartile range; codes ICD-10: J00 – acute nasopharyngitis (cold), J04 – acute laryngitis and tracheitis, J10 and J11 – influenza, J12–J18 – pneumonia, J20–J21 – acute bronchitis and/or bronchiolitis, J45–J46 – asthma exacerbation or status asthmaticus.

### A relationship between the daily number of MS due to respiratory diseases and the worsening the quality of ambient air

Next, the results obtained using multivariate analyses confirmed a relationship between daily number of MS due to respiratory diseases and worsening the quality of ambient air in Gliwice city in the studied period.

**Fig 3:**
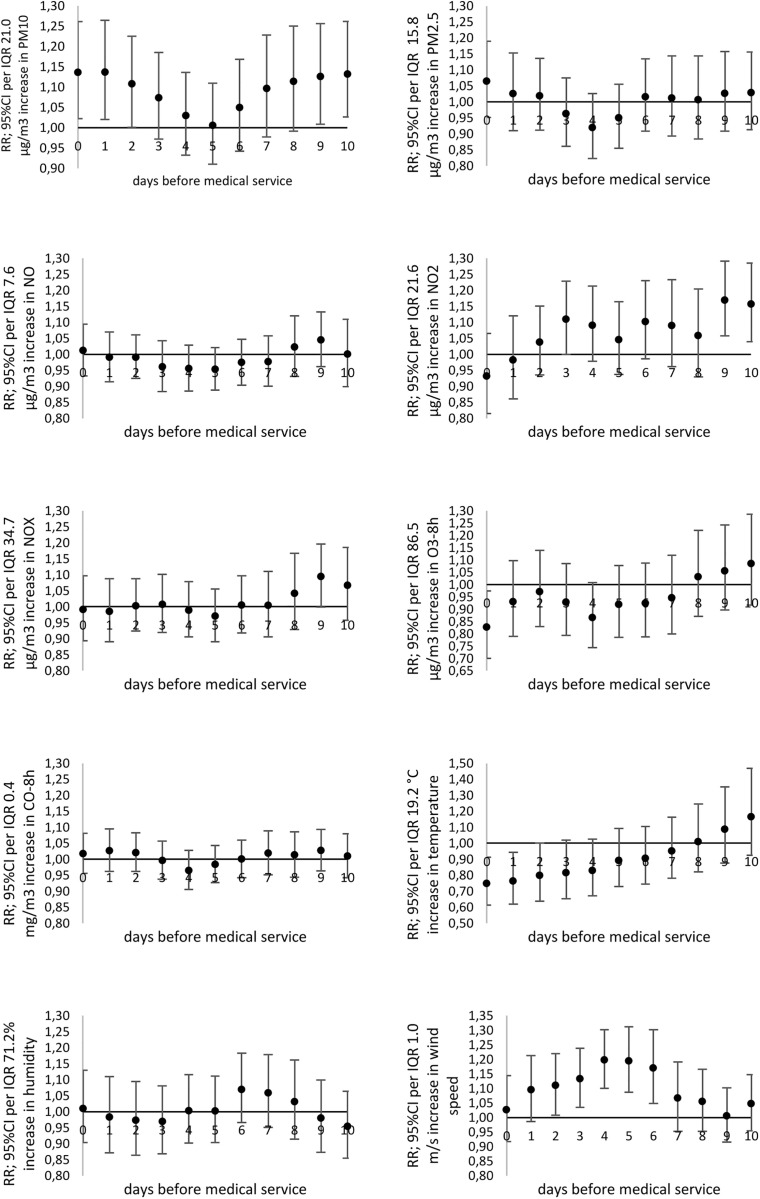
The relative risk (*RR*) of medical services (MS) due to acute nosopharyngitis (cold) (J00; ICD-10) related to particular pollutants or meteorological factors increases by the *IQR* value.

**Fig 4:**
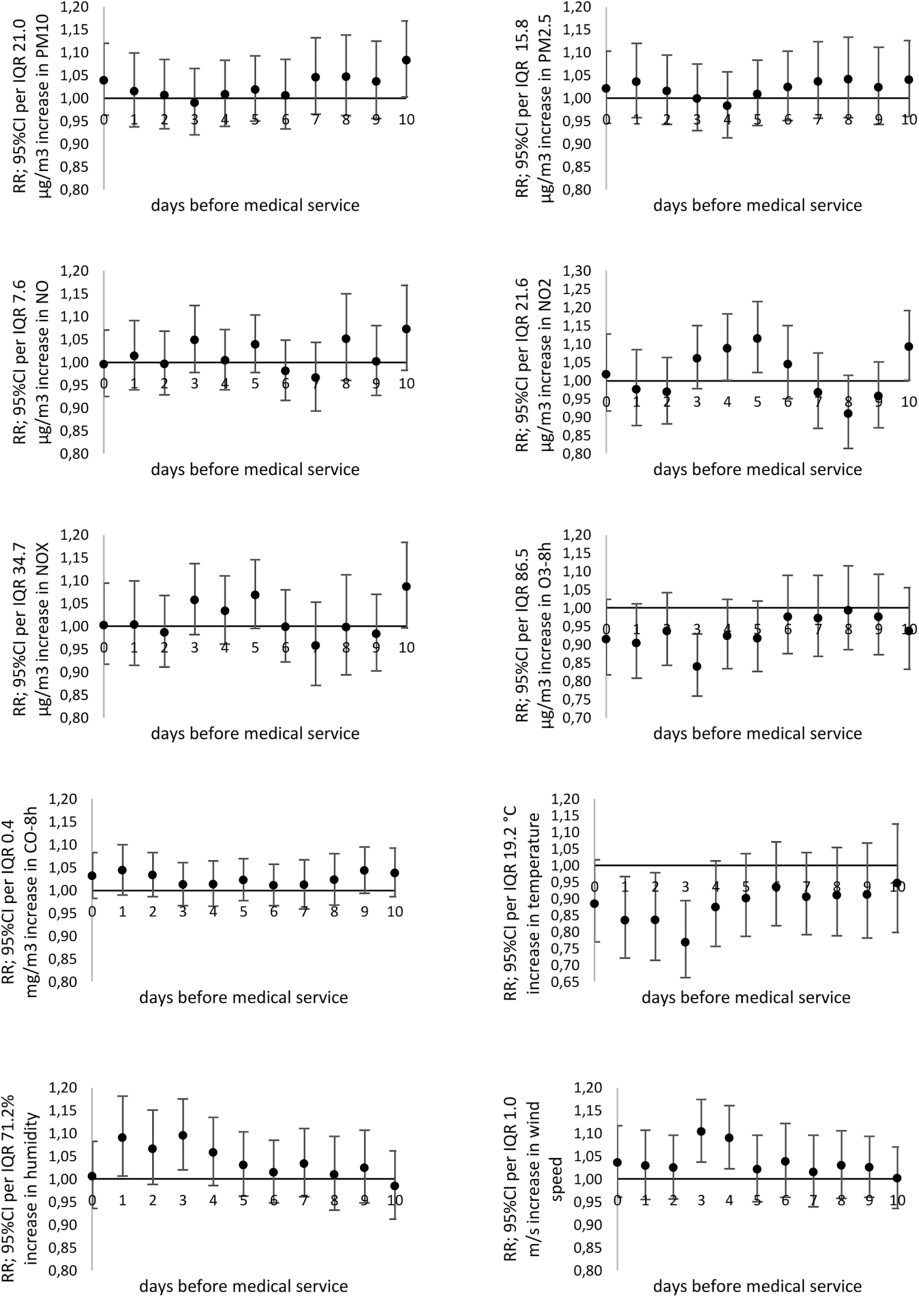
The relative risk (*RR*) of medical services (MS) due to asthma (J45-J46; ICD-10) related to particular pollutants or meteorological factors increases by the *IQR* value.

A significant increase in the risk of daily MS due to acute nasopharyngitis (J00) was observed in response to PM_10_ (RR_max_ = 1.137; 95% CI: 1.020-1.264 with 2-day lag), NO_2_ (RR_max_ = 1.169; 95% CI: 1.057-1.291 with 9-day lag) (Fig 3). An increase in 8-hour ozone concentration (O_3_-8h) by the unit led to a significantly lower risk of MS due to acute nasopharyngitis (J00), laryngitis and tracheitis (J04), bronchitis (J20-J21), and asthma exacerbation (J45-J46) (Figs 3 and 4, S1 and S2 Figs).

It was observed that a significant increase in the risk of daily MS due to laryngitis and tracheitis (J04) occurred with the 1-day lag of exposition to PM_10_ (RR_max_ = 1.164; 95% CI: 1.051-1.288), as well as PM_2.5_ (RR_max_ = 1.123; 95% CI: 1.029-1.223 with 1-day lag) (S1 Fig).

There was no observed relationship between influenza (J10-J11) incidents and ambient air quality conditions during the study period.

**Fig 5:**
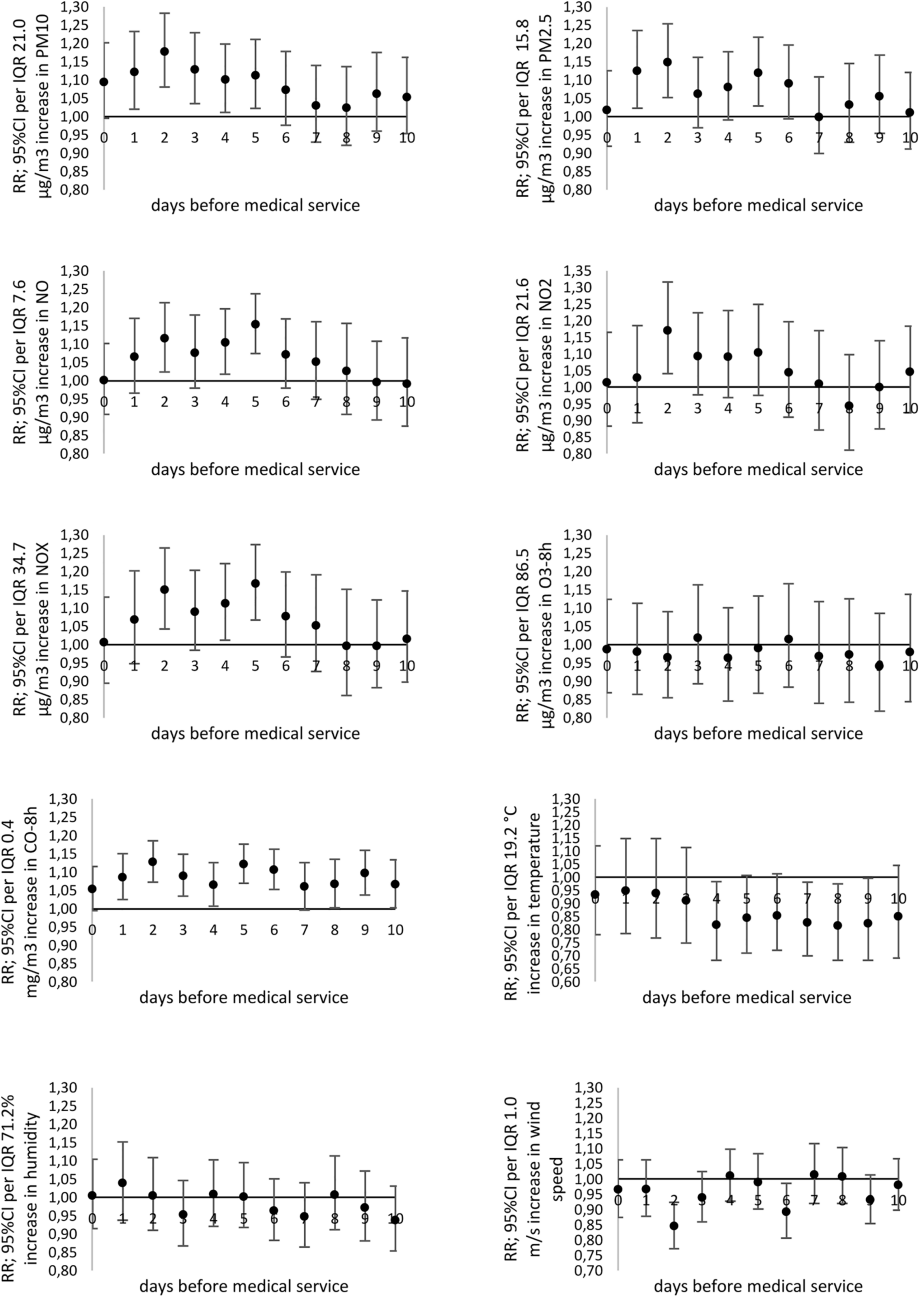
The relative risk (*RR*) of medical services (MS) due to pneumonia (J12-J18; ICD-10) related to particular pollutants or meteorological factors increases by the *IQR* value.

The highest risk of daily MS due to pneumonia (J12-J18) was noted for PM_10_ exposure (RR_max_ = 1.178; 95% CI: 1.081-1.283 with 2-day lag), and PM_2.5_ (RR_max_ = 1.149; 95% CI: 1.070-1.232 with 2-day lag). In the case of nitrogen oxides, significant relative risk was noted with a 2-day lag. Moreover, the highest risk of pneumonia was related to the increase in concentration of NO (RR_max_ = 1.154; 95% CI: 1.074-1.238 with 5-day lag); NO_2_ (RR_max_ = 1.171; 95% CI: 1.040-1.316 with 2-day lag); and NO_X_ (RR_max_ = 1.167; 95% CI: 1.067-1.274 with 5-day lag). Detailed results are presented in Fig 5.

In the case of bronchitis (J20-J21) relative risk of MS significantly increased with NO_2_ increasing exposure over a longer time with the maximum value of 10 days lag (RR_max_ = 1.122; 95% CI: 1.009-1.247) (S2 Fig).

However, in the case of asthma exacerbation (J45-J46), an increase in relative risk of medical services was observed for PM_10_ exposure and the 10-day delay (RR_max_ = 1.083; 95%CI: 1.003-1.169), and NO_2_ (RR_max_ = 1.115; 95%CI: 1.022-1.217 for 5-day delay) (Fig 4).

### A relationship between the daily number of MS due to respiratory diseases and meteorological conditions

An increase in ambient air temperature by the unit led to a significantly lower risk of MS due to acute nasopharyngitis (J00), laryngitis and tracheitis (J04), pneumonia (J12-J18), bronchitis (J20-J21), and asthma exacerbation (J45-J46) (Figs 3–5, S1 and S2 Figs).

Moreover, it has been shown that the increase in wind speed was associated with a higher relative risk of MS due to acute nasopharyngitis (J00) (RR_max_ = 1.197; 95% CI: 1.100-1.301 for 4-day lag), laryngitis and tracheitis (J04) (RR_max_ = 1.162; 95% CI: 1.058-1.275 for 5-day lag), bronchitis (J20-J21) (RR_max_ = 1.131; 95% CI: 1.044-1.224 with 4-day lag), asthma (J45-J46) (RR_max_ = 1.104; 95%CI: 1.037-1.174 and 3-day delay) (Fig 3, 4, S2). The increase in wind speed significantly reduced the daily number of medical services due to pneumonia (J12-J18) (Fig 5). Additionally, an increase in relative air humidity just before an asthma exacerbation (1−3 days prior) was significantly associated with the risk of MS (RR_max_ = 1.095; 95%CI: 1.020-1.176 with 3-day lag).

### Comparison of models

The second model of multivariate analysis which was used to assess the relative risk of daily medical services due to respiratory diseases confirmed that higher concentrations of average moving concentration of ambient air pollutants were related to the higher relative risk of daily MS due to acute respiratory incidents. Tables 3 and S3 present detailed results for selected diseases respectively.

**Table 3:** The risk ratio (RR) of medical services (MS) due to selected respiratory diseases related to particular pollutants or meteorological factors increases by the unit or 10 units.

ICD-10		J00										J12-J18										J45-J46									
Moving average		daily	2-day	3-day	4-day	5-day	6-day	7-day	8-day	9-day	10-day	daily	2-day	3-day	4-day	5-day	6-day	7-day	8-day	9-day	10-day	daily	2-day	3-day	4-day	5-day	6-day	7-day	8-day	9-day	10-day
RR and 95%CI per 10 units/ 1 unit* increase	**PM**_**10**_ **[µg/m** ^**3**^ **]**	1.076 (1.013;1.142)	1.092 (1.022;1.166)	1.102 (1.027;1.181)	1.103 (1.025;1.186)	1.094 (1.014;1.179)	1.083 (1.002;1.17)	1.082 (1;1.171)	1.091 (1.005;1.184)	1.105 (1.013;1.203)	1.121 (1.025;1.226)	1.053 (0.997;1.111)	1.071 (1.01;1.134)	1.103 (1.038;1.171)	1.118 (1.05;1.189)	1.126 (1.055;1.201)	1.134 (1.062;1.211)	1.132 (1.058;1.211)	1.131 (1.053;1.212)	1.134 (1.052;1.22)	1.142 (1.056;1.234)	1.022 (0.979;1.067)	1.018 (0.97;1.068)	1.013 (0.962;1.067)	1.007 (0.953;1.063)	1.006 (0.95;1.064)	1.005 (0.948;1.065)	1.002 (0.945;1.063)	1.005 (0.946;1.068)	1.008 (0.945;1.074)	1.008 (0.942;1.078)
	**PM**_**2.5**_ **[µg/m** ^**3**^ **]**	1.068 (0.962;1.182)	1.062 (0.943;1.195)	1.059 (0.932;1.201)	1.047 (0.914;1.197)	0.996 (0.867;1.142)	0.975 (0.847;1.121)	0.974 (0.837;1.131)	0.98 (0.831;1.153)	0.974 (0.816;1.159)	0.979 (0.815;1.172)	1.019 (0.923;1.123)	1.115 (1;1.241)	1.201 (1.076;1.339)	1.191 (1.062;1.334)	1.194 (1.064;1.339)	1.237 (1.102;1.389)	1.265 (1.115;1.433)	1.279 (1.114;1.466)	1.289 (1.113;1.491)	1.301 (1.118;1.511)	1.023 (0.942;1.109)	1.039 (0.946;1.139)	1.033 (0.936;1.138)	1.028 (0.931;1.135)	1.023 (0.925;1.13)	1.008 (0.91;1.115)	1.012 (0.905;1.129)	1.014 (0.898;1.144)	1.019 (0.895;1.158)	1.016 (0.889;1.16)
	**NO** **[µg/m** ^**3**^ **]**	1.012 (0.932;1.095)	1 (0.901;1.108)	0.987 (0.857;1.136)	0.96 (0.801;1.148)	0.908 (0.749;1.095)	0.877 (0.723;1.059)	0.868 (0.713;1.052)	0.86 (0.695;1.06)	0.852 (0.678;1.065)	0.892 (0.708;1.119)	1.002 (0.907;1.102)	1.052 (0.929;1.186)	1.182 (1.014;1.374)	1.274 (1.066;1.52)	1.365 (1.143;1.625)	1.523 (1.292;1.791)	1.547 (1.306;1.829)	1.619 (1.351;1.937)	1.653 (1.356;2.013)	1.607 (1.307;1.971)	0.996 (0.925;1.071)	1.006 (0.914;1.105)	1.001 (0.882;1.135)	1.062 (0.915;1.232)	1.066 (0.913;1.24)	1.084 (0.932;1.258)	1.067 (0.913;1.243)	1.043 (0.882;1.231)	1.067 (0.891;1.274)	1.066 (0.889;1.275)
	**NO**_**2**_ **[µg/m** ^**3**^ **]**	0.945 (0.848;1.052)	0.944 (0.83;1.072)	0.965 (0.832;1.119)	1.04 (0.883;1.224)	1.073 (0.907;1.266)	1.095 (0.925;1.295)	1.13 (0.958;1.332)	1.163 (0.983;1.374)	1.173 (0.985;1.397)	1.23 (1.033;1.463)	1.012 (0.905;1.13)	1.021 (0.897;1.161)	1.114 (0.966;1.283)	1.143 (0.985;1.325)	1.157 (0.992;1.346)	1.177 (1.01;1.37)	1.151 (0.989;1.339)	1.134 (0.972;1.321)	1.113 (0.947;1.308)	1.11 (0.942;1.307)	1.014 (0.933;1.101)	0.993 (0.9;1.094)	0.973 (0.871;1.085)	1.019 (0.909;1.141)	1.069 (0.952;1.2)	1.102 (0.982;1.235)	1.111 (0.994;1.242)	1.101 (0.984;1.232)	1.082 (0.962;1.217)	1.069 (0.948;1.204)
	**NO**_**X**_ **[µg/m** ^**3**^ **]**	0.997 (0.957;1.037)	0.991 (0.943;1.041)	0.989 (0.929;1.053)	0.996 (0.925;1.073)	0.99 (0.916;1.068)	0.985 (0.911;1.064)	0.991 (0.916;1.071)	1 (0.919;1.086)	1.001 (0.915;1.094)	1.024 (0.935;1.12)	1.003 (0.957;1.049)	1.02 (0.964;1.078)	1.072 (1.004;1.143)	1.094 (1.019;1.173)	1.113 (1.037;1.195)	1.147 (1.072;1.228)	1.144 (1.067;1.227)	1.149 (1.067;1.237)	1.151 (1.061;1.247)	1.145 (1.053;1.244)	1.001 (0.967;1.036)	1 (0.958;1.044)	0.993 (0.942;1.047)	1.018 (0.961;1.078)	1.031 (0.973;1.092)	1.043 (0.984;1.103)	1.042 (0.984;1.103)	1.037 (0.976;1.101)	1.038 (0.972;1.108)	1.034 (0.967;1.105)
	**O**_**3**_ **-8h** **[µg/m** ^**3**^ **]**	0.962 (0.929;0.995)	0.969 (0.933;1.005)	0.974 (0.936;1.014)	0.973 (0.934;1.014)	0.967 (0.927;1.009)	0.966 (0.925;1.009)	0.966 (0.924;1.011)	0.965 (0.922;1.011)	0.967 (0.921;1.015)	0.97 (0.922;1.021)	0.998 (0.971;1.024)	0.998 (0.969;1.027)	0.996 (0.965;1.027)	0.997 (0.962;1.033)	0.994 (0.957;1.032)	0.998 (0.959;1.038)	1 (0.96;1.042)	1.001 (0.96;1.045)	1.003 (0.959;1.048)	1.001 (0.956;1.049)	0.982 (0.959;1.005)	0.976 (0.951;1.001)	0.972 (0.946;0.999)	0.96 (0.933;0.987)	0.957 (0.929;0.985)	0.955 (0.926;0.984)	0.956 (0.927;0.987)	0.956 (0.926;0.987)	0.954 (0.923;0.986)	0.95 (0.917;0.984)
	**CO-8h** **[mg/m** ^**3**^ ]*	1.091 (0.799;1.478)	1.144 (0.803;1.617)	1.175 (0.802;1.716)	1.139 (0.755;1.715)	1.045 (0.676;1.609)	1.004 (0.648;1.549)	1.003 (0.647;1.548)	1.023 (0.651;1.598)	1.033 (0.648;1.636)	1.06 (0.659;1.692)	1.303 (0.974;1.732)	1.506 (1.089;2.071)	1.94 (1.38;2.723)	2.207 (1.534;3.175)	2.282 (1.559;3.339)	2.513 (1.729;3.65)	2.616 (1.805;3.788)	2.598 (1.769;3.812)	2.624 (1.765;3.896)	2.715 (1.819;4.043)	1.171 (0.914;1.492)	1.265 (0.952;1.673)	1.336 (0.981;1.815)	1.34 (0.96;1.868)	1.341 (0.944;1.9)	1.341 (0.946;1.897)	1.314 (0.928;1.854)	1.315 (0.92;1.873)	1.337 (0.925;1.927)	1.386 (0.954;2.005)
	**Temperature** **[°C]**	0.727 (0.585;0.904)	0.713 (0.564;0.901)	0.703 (0.545;0.905)	0.691 (0.527;0.906)	0.679 (0.511;0.902)	0.686 (0.511;0.92)	0.698 (0.518;0.942)	0.717 (0.53;0.972)	0.742 (0.544;1.012)	0.774 (0.562;1.064)	0.928 (0.761;1.132)	0.928 (0.75;1.15)	0.922 (0.731;1.163)	0.91 (0.71;1.166)	0.868 (0.67;1.126)	0.841 (0.645;1.099)	0.823 (0.629;1.078)	0.801 (0.611;1.052)	0.781 (0.592;1.031)	0.767 (0.577;1.019)	0.874 (0.751;1.018)	0.833 (0.708;0.981)	0.801 (0.67;0.957)	0.749 (0.621;0.904)	0.734 (0.603;0.894)	0.726 (0.593;0.889)	0.733 (0.598;0.899)	0.73 (0.594;0.897)	0.722 (0.585;0.892)	0.711 (0.572;0.884)
	**Humidity** **[%]**	1.006 (0.933;1.086)	0.997 (0.914;1.088)	0.988 (0.898;1.087)	0.979 (0.886;1.082)	0.981 (0.885;1.087)	0.982 (0.883;1.092)	0.998 (0.895;1.113)	1.008 (0.899;1.13)	1.013 (0.896;1.143)	1.007 (0.882;1.149)	1.003 (0.941;1.07)	1.018 (0.946;1.096)	1.016 (0.938;1.102)	0.998 (0.916;1.086)	0.998 (0.913;1.09)	0.997 (0.908;1.093)	0.985 (0.895;1.085)	0.972 (0.878;1.076)	0.971 (0.871;1.082)	0.961 (0.854;1.08)	1.004 (0.956;1.055)	1.037 (0.98;1.098)	1.056 (0.991;1.124)	1.078 (1.01;1.151)	1.086 (1.015;1.162)	1.091 (1.016;1.171)	1.091 (1.014;1.174)	1.098 (1.018;1.185)	1.106 (1.02;1.199)	1.124 (1.029;1.227)
	**Wind Speed** [m/s]*	1.053 (0.842;1.309)	1.214 (0.923;1.589)	1.372 (1.021;1.831)	1.528 (1.132;2.051)	1.806 (1.337;2.43)	2.112 (1.546;2.875)	2.359 (1.698;3.264)	2.551 (1.783;3.635)	2.802 (1.902;4.109)	2.72 (1.801;4.085)	0.933 (0.766;1.13)	0.896 (0.699;1.143)	0.694 (0.524;0.914)	0.658 (0.488;0.881)	0.688 (0.503;0.936)	0.679 (0.486;0.945)	0.609 (0.427;0.864)	0.606 (0.414;0.882)	0.596 (0.396;0.89)	0.537 (0.352;0.815)	1.073 (0.922;1.246)	1.116 (0.92;1.35)	1.164 (0.939;1.437)	1.337 (1.074;1.659)	1.467 (1.171;1.831)	1.506 (1.183;1.911)	1.563 (1.211;2.012)	1.623 (1.234;2.127)	1.731 (1.291;2.313)	1.819 (1.339;2.46)

*RR (95%CI)*, Relative Risk and 95% Confidence Interval; MS – medical services; codes ICD-10: J00 – acute nasopharyngitis (cold), J12–J18 – pneumonia, J45–J46 – asthma exacerbation or status asthmaticus.

*increase by one unit.

Both models showed similar results; higher concentration of ambient air pollution increased the risk of daily MS – Table 4.

**Table 4:** Summary of significant results of multivariate analysis models, performed according to two scenarios, in the form of maximum values *RR*_*max*_ of relative risk values taking into account the delayed health effect.

ICD-10 code	Air pollutants and meteorological factors	1 scenario		2 scenario	
		*RR*_*max*_ *(95%CI)*	*l*-days before MS	*RR*_*max*_ *(95%CI)*	Average *l*-days before MS
J00	PM_10_	1.137 (1.020-1.264)	2	1.121 (1.025-1.226)	10
	NO_2_	1.169 (1.057-1.291)	9	1.230 (1.033-1.463)	10
	Wind speed	1.197 (1.100-1.301)	4	2.802 (1.902-4.109)	9
J04	PM_10_	1.164 (1.051-1.288)	1	–	–
	PM_2.5_	1.123 (1.029-1.223)	1	1.140 (1.009-1.285)	3
	Wind speed	1.162 (1.058-1.275)	5	1.887 (1.214-2.916)	10
J12-J18	PM_10_	1.178 (1.081-1.283)	2	1.142 (1.056-1.234)	10
	PM_2.5_	1.149 (1.070-1.232)	2	1.301 (1.118-1.511)	10
	NO	1.154 (1.074-1.238)	5	1.653 (1.356-2.013)	9
	NO_2_	1.171 (1.040-1.316)	2	–	–
	NO_X_	1.167 (1.067-1.274)	5	1.151 (1.061-1.247)	9
J20-J21	NO_2_	1.122 (1.009-1.247)	10	–	–
	Wind speed	1.131 (1.044-1.224)	4	1.970 (1.358-2.847)	9
J45-J46	PM_10_	1.083 (1.003-1.169)	10	–	–
	NO_2_	1.115 (1.022-1.217)	5	–	–
	Humidity	1.095 (1.020-1.176)	3	1.124 (1.029-1.227)	10
	Wind speed	1.104 (1.037-1.174)	3	1.819 (1.339-2.460)	10

1 scenario – delays of MS, **l* *= 1, 2, 3,..., 10 days; 2 scenario – moving average concentrations of pollutants, **l* *= 1, 2, 3,..., 10 days preceding the MS; MS – medical services; *RR*_*max*_ *(95%CI)*, maximum value of Relative Risk and 95% Confidence Interval; codes ICD-10: J00 – acute nasopharyngitis (cold), J04 – acute laryngitis and tracheitis, J12–J18 – pneumonia, J20–J21 – acute bronchitis and/or bronchiolitis, J45–J46 – asthma exacerbation or status asthmaticus.

## Discussion

In the warm season of the 2024 year, the highest number of medical services due to acute respiratory diseases included nasopharyngitis J00 (n = 8,329; 17.0%) and asthma J45-J46 (n = 4,917; 10.0%), mostly in children aged 0–9 years. This observation is in line with previous results obtained for children and adolescents hospitalized due to asthma in Poland [32]. Our study confirmed a statistically significant relationship between ambient air quality deterioration and an increase in the number of MS due to acute respiratory problems in residents of Gliwice city. In calculations of risk ratio, we used two variants of exposure (increase in concentrations of air pollutants by the interquartile range value or increase in concentration by 10 µg/m^3^, and for carbon monoxide or wind speed, the increase by 1 unit). Both models brought similar results showing that higher concentration of ambient air pollution increased the risk of daily MS. However, it should be emphasized that the observed relationships are suggestive and not definitive due to the study design and its ecological nature.

### Pollutant-specific effects

In the case of acute nasopharyngitis (J00), laryngitis and tracheitis (J04), pneumonia (J12-J18) the highest risk of the mentioned health problem was obtained for a longer time of fine particle exposure and nitrogen oxides. This observation is consistent with the well-documented knowledge of health delays related to air pollution exposure [14,23–25]. The level of health consequences and the proportion of people affected by particular diseases are significantly related to time of exposure [33]. Initially, directly after exposure symptoms in the upper respiratory tract were recognized, next longer time of exposure and the ongoing inflammatory process can lead to consequences in the lower respiratory tract, including pneumonia and bronchitis [34,35]. A stronger effect can affect children and older people with weaker immunosystems or comorbidities.

There was no relationship observed between influenza incidents and ambient air quality conditions during the study period. This lack of correlation is evident, particularly because the improvement in air quality was attributed to natural ventilation. This finding aligns with discussions in previous studies focused on winter smog [36].

It was confirmed that a significant increase in the relative risk of medical services due to bronchitis (J20-J21) was linked to higher nitrogen dioxide (NO_2_) exposure. This observation aligns with previous data on NO_X_ exposure in the Silesian Voivodeship population, as well as bronchitis and asthma exacerbations [9].

The increase in the risk of medical services related to asthma (J45-J46) was associated with longer exposure to PM_10_. In contrast, the impact of NO_2_ was observed sooner.

In summary, while the concentrations of particulate matter in the atmosphere are considerably lower during the warmer months compared to winter, our study results show that an increase in these pollutants significantly raises the risk of acute respiratory incidents among the residents of Gliwice. Acute pharyngitis, rhinitis, laryngitis, and tracheitis can develop shortly after exposure, sometimes even on the first day. However, the highest risk of pneumonia is linked to a longer period of exposure, which can last up to 10 days. The significant impact of nitrogen oxide exposure is evident, and for the observed medical services, we experience a delayed effect of up to 9–10 days.

### Meteorological conditions

An increase in ambient air temperature or 8-hour ozone concentration (O_3_-8h) was related to the lower risk of MS due to J00. This observation is in line with the results of a previous study in the same region and warmer season of the year, where the survival of microorganisms (including pathogenic ones) is lower [37,38]. Therefore, a lower concentration of bioaerosols measured in ambient air is probably related to a lower risk of developing acute throat or nasal diseases.

The obtained results confirmed, that an increase in wind speed causes a lower number of medical services due to pneumonia. This is evident due to the improved air quality resulting from natural ventilation, which has been consistently highlighted in previous studies related to winter smog [39,40].

Additionally, an increase in relative air humidity just before an asthma exacerbation (1–3 days prior) was significantly associated with the risk of MS. As we previously mentioned, higher temperatures and relative humidity favor the survival of fungi [37]. The published data confirm that fungi in ambient air can be linked to the development and worsening of asthma, particularly in children [41–43]. Our results indicate that an increase in wind speed is associated with a higher risk of asthma exacerbations. That observation is in line with the current evidence regarding the impact of severe meteorological phenomena, such as high relative humidity, strong wind, sudden weather changes, low or high temperatures, rain, wind, and storms, on asthma exacerbation [44]. The results of a study conducted in a Chinese city suggest that meteorological factors, including changes in atmospheric pressure and the accompanying increase in wind speed, are more strongly associated with asthma exacerbation than air pollution [45]. Another study from China confirmed a link between increased hospitalizations due to asthma in younger patients (aged 19 years or younger) and those over 60 years old, associated with low humidity and higher wind speeds [46].

Meteorological conditions typical for the warm season of the year, high temperatures, and sunlight also significantly affected the risk of MS due to pharyngitis and rhinitis, laryngitis and tracheitis, bronchitis, and asthma exacerbation. The risk of mentioned health effects decreases as meteorological parameters increase. On the other hand, higher relative humidity increases the risk of asthma exacerbation and bronchitis. Similar results were noted for outpatient visits due to acute upper respiratory infections in children under 15 in Taipei, Taiwan [47]. The impact of a one-unit increase in wind speed on the risk of medical services due to pharyngitis, rhinitis, laryngitis, tracheitis, bronchitis, bronchiolitis, and asthma exacerbation is challenging to assess. On the one hand, available published data suggest the opposite effect; lower wind speeds are typically associated with an increased risk of severe acute respiratory infections [48]. On the other hand, some data revealed that an increase in wind speed was related to the increase in the number of respiratory incidents, i.e., acute upper respiratory infections or diseases of the upper and lower respiratory tracts [42,43,49]. The authors of these works highlight that the mechanism of this interaction is not fully understood and requires further studies and explanations [45].

However, discussing the results, climatic and environmental differences should be taken into account. The city of Gliwice has a temperate climate, and many studies on summer exposure were mainly conducted in large cities that are prone to developing summer smog.

### Limitations of the study

The applied research model (ecological study), combined with the lack of individual exposure data and a relatively small number of recorded acute respiratory incidents per day, means that the results presented should be interpreted with great caution.

It should be noted that ecological studies limit conclusions at the individual level. The data provided by the National Health Fund included the total number of medical services provided per day. They were completely anonymous and did not include information on the patients’ place of residence. Therefore, it was not possible to attribute meteorological data or air pollution levels to specific spatial locations. This made it impossible to assess individual risk.

Furthermore, errors associated with accurately recording the reasons for the provided medical services cannot be excluded. It should also be emphasized that only medical services related to the primary disease were taken into account, excluding those associated with multimorbidity.

Next, the relatively short observation period (according to the seasonal classification relevant to southern Poland’s temperate climate, “summer” encompasses July and August, and “early autumn” refers to September and October), restrict the precision of the conclusions drawn. It is also worth mentioning the high climatic variability over the years, which may disturb the correct interpretation of the results. A long-term study should take into account the cool and cold seasons, during which Poland experiences a high incidence of respiratory illnesses. These periods are also characterized by elevated levels of air pollution. Such research assumptions lead to conclusions focusing on severe exposure. The authors’ intention was to focus on the influence of environmental factors present in the summer environment.

The limitation is also the small population, limited to residents of a single city (part of a large agglomeration). Presented findings may not be generalizable to other regions with different pollution profiles or health infrastructure.

Additionally, a limitation of the study is the statistical model used to estimate the cause-and-effect relationship, which did not simultaneously account for meteorological factors as potential confounders. These factors are known to have a significant and strong correlation with air pollution levels. Furthermore, the data on patient age were provided in aggregated form, making it impossible to conduct adjusted analyses.

### Public health

Although there are limitations noted, the findings on the link between ambient air quality in summer and the incidence of medical services due to acute respiratory events are the first data of this kind obtained for Poland. Ongoing climate changes have led to an increasing frequency of heat waves in our country [50]. Along with this trend, there is a growing social demand for reliable information about environmental health risks. Therefore, we believe it is important to present the results of our research, which aims to highlight the need for initiating corrective actions. It is also necessary to conduct similar studies in other centers to obtain more in-depth conclusions.

This evidence can play a key role in effectively communicating environmental health risks, educating the public about potential dangers, and putting pressure on policymakers responsible for legislation and risk management.

In practical terms, it can support local air-quality management and early warning systems in warmer summers by defining health-based thresholds for public advisories during forecast atmospheric stagnation or heatwaves and by integrating air-quality with short-range meteorological forecasts. It can also guide targeted risk communication to vulnerable groups (e.g., children, older adults, and people with asthma or chronic obstructive pulmonary disease [COPD]) and inform healthcare preparedness planning in primary care and emergency departments.

It is also an important element of environmental management. Monitoring air quality parameters in relation to population exposure in urban areas is essential for effective environmental risk management and should be a fundamental component of environmental planning [51–53].

## Conclusion

The study results indicate that higher levels of ambient air pollution, specifically from particulate matter and nitrogen oxides, significantly impact the increase in daily medical services due to acute respiratory incidents. Additionally, the research highlighted the independent importance of meteorological conditions, such as wind speed and relative humidity, in this relationship. Longer times of exposure are associated with a greater risk; however, statistically significant effects related to particulate matter (PM_10_ and PM_2.5_) can also be observed shortly after exposure, within a moving average concentration of 1–2 days. Research on how weather affects the risk of acute respiratory incidents in the warm season needs further long-term studies on larger populations.

The results play a crucial role in effectively communicating environmental health risks, educating the public about potential threats, and applying pressure on politicians responsible for legislation and risk management.

## Supporting information

S1 Table Number of registered medical services (MS) provided to patients diagnosed with J00-J99 in Gliwice, by age groups (period 01/07/2024–31/10/2024). (TIF)

S2 Table Number of registered medical services (MS) in Gliwice, including the main diagnosis of the service, and the age groups of patients. (TIF)

S3 Table The relative risk (RR) of medical services (MS) due to selected respiratory diseases, related to particular pollutants or meteorological factors increases by the unit or 10 units. (TIF)

S1 Fig The relative risk (RR) of medical services (MS) due to acute laryngitis and tracheitis (J04; ICD-10) related to particular pollutants or meteorological factors increases by the IQR value. (TIF)

S2 Fig The relative risk (RR) of medical services (MS) provided due to acute bronchitis and/or bronchiolitis (J20-J21; ICD-10) related to particular pollutants or meteorological factors increases by the IQR value. (TIF)
