# Supplementary figures and images for: Respiratory incidents in response to air quality deterioration in the summer and early autumn season
Source: PLoS One. 2025 Oct 22;20(10):e0335063. doi: 10.1371/journal.pone.0335063 (PMC12543195; doi:10.1371/journal.pone.0335063)

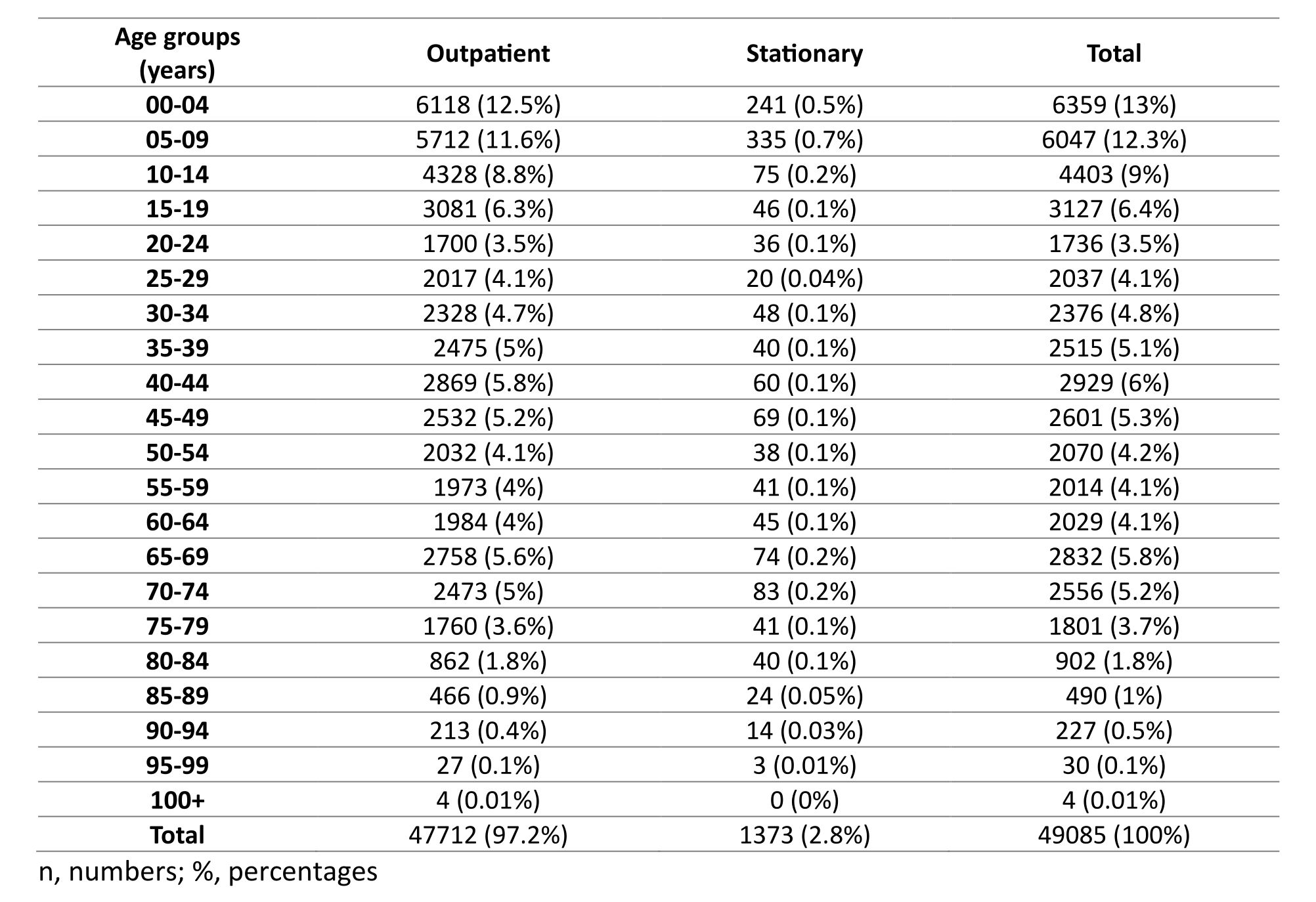

Supplement: S1 Table — (TIF) [file pone.0335063.s001.tif]

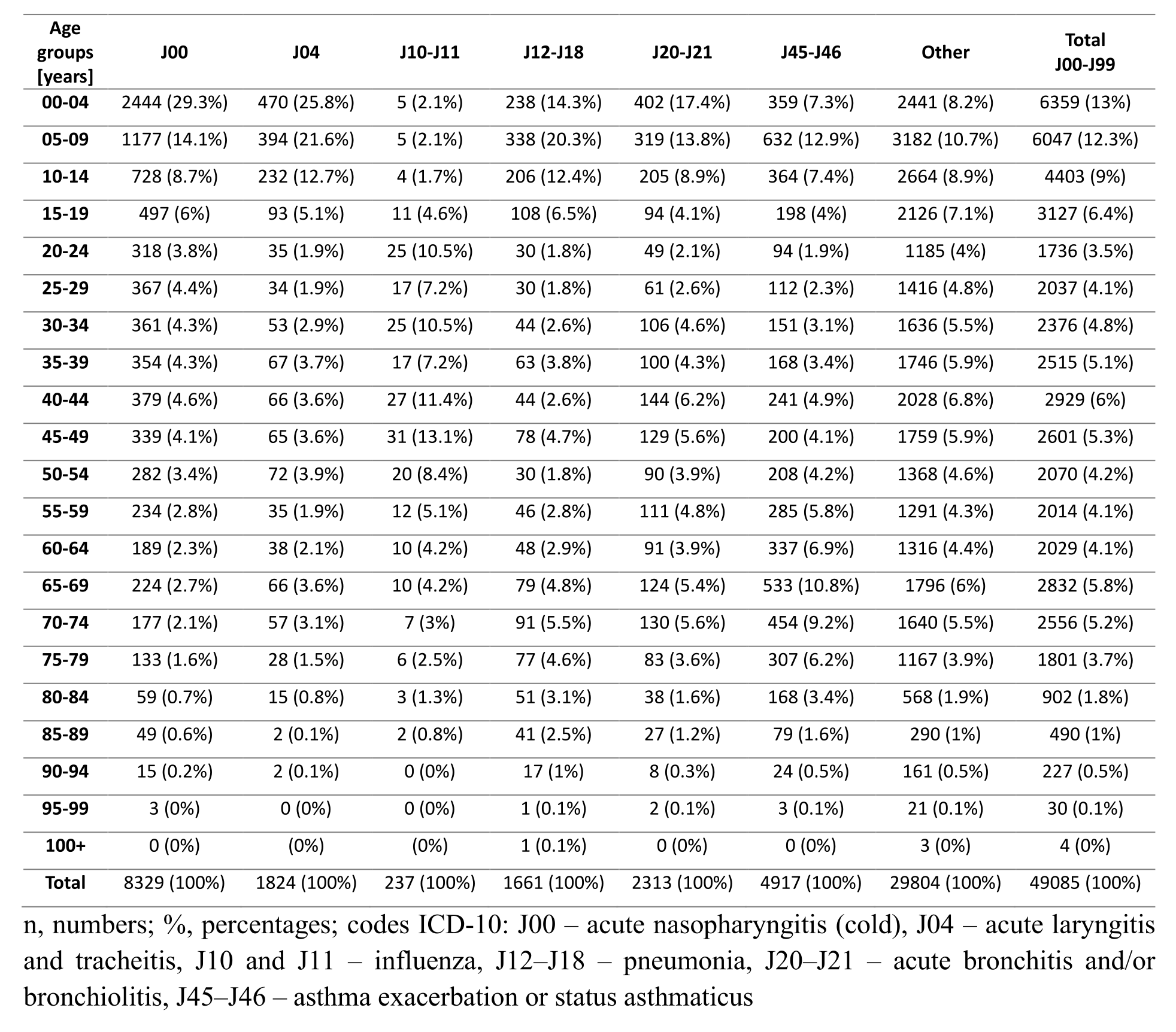

Supplement: S2 Table — (TIF) [file pone.0335063.s002.tif]

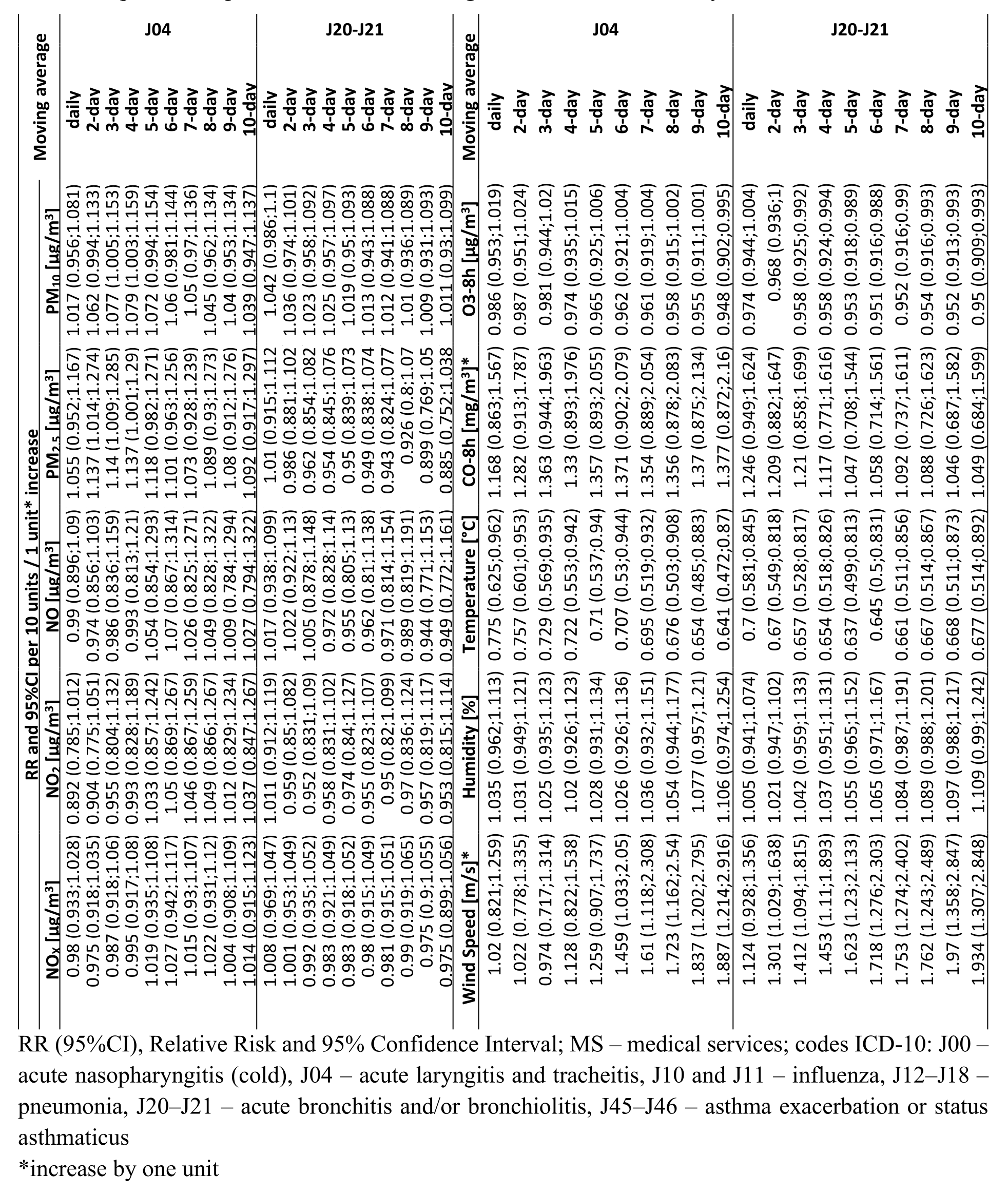

Supplement: S3 Table — (TIF) [file pone.0335063.s003.tif]

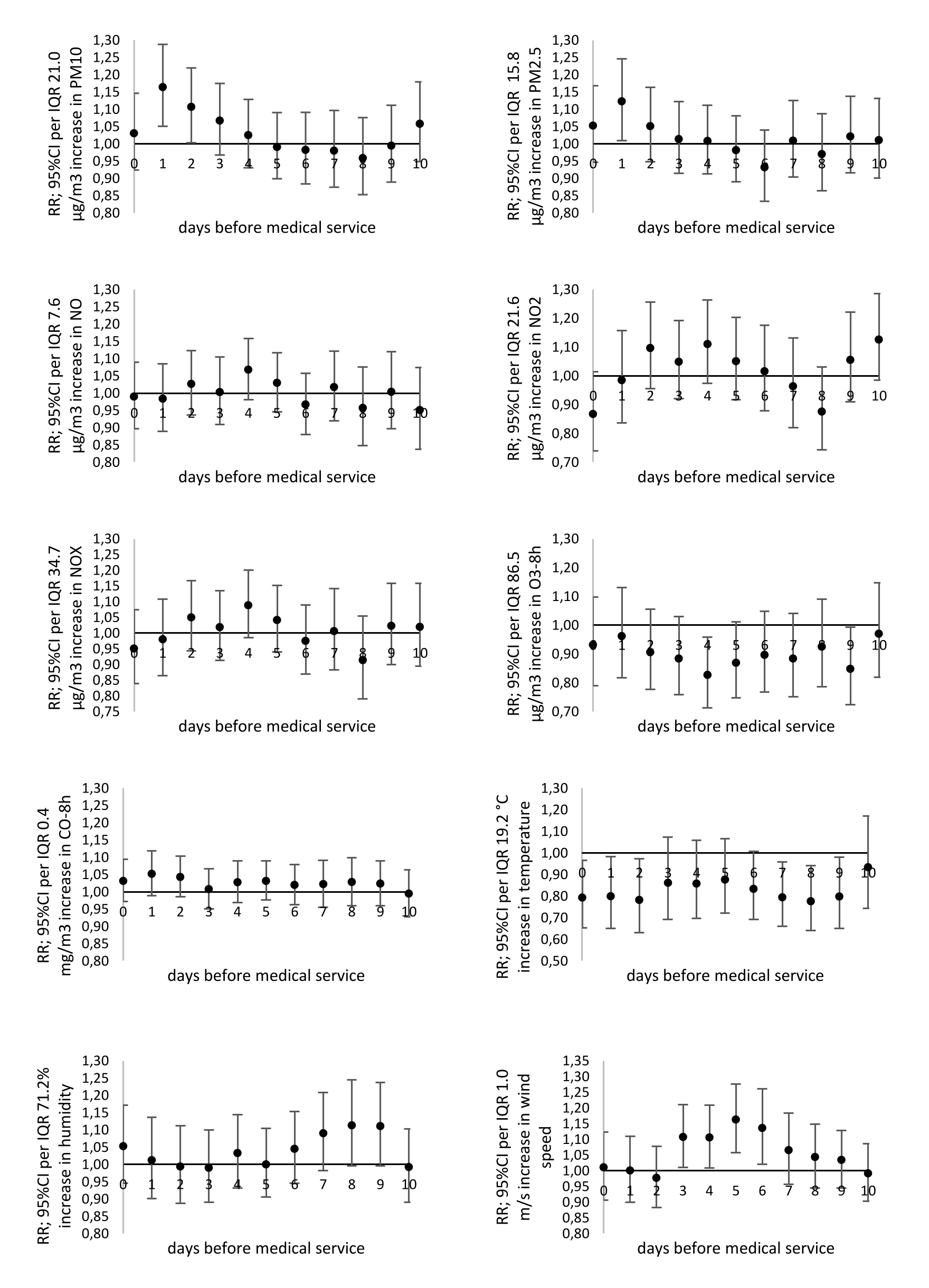

Supplement: S1 Fig — (TIF) [file pone.0335063.s004.tif]

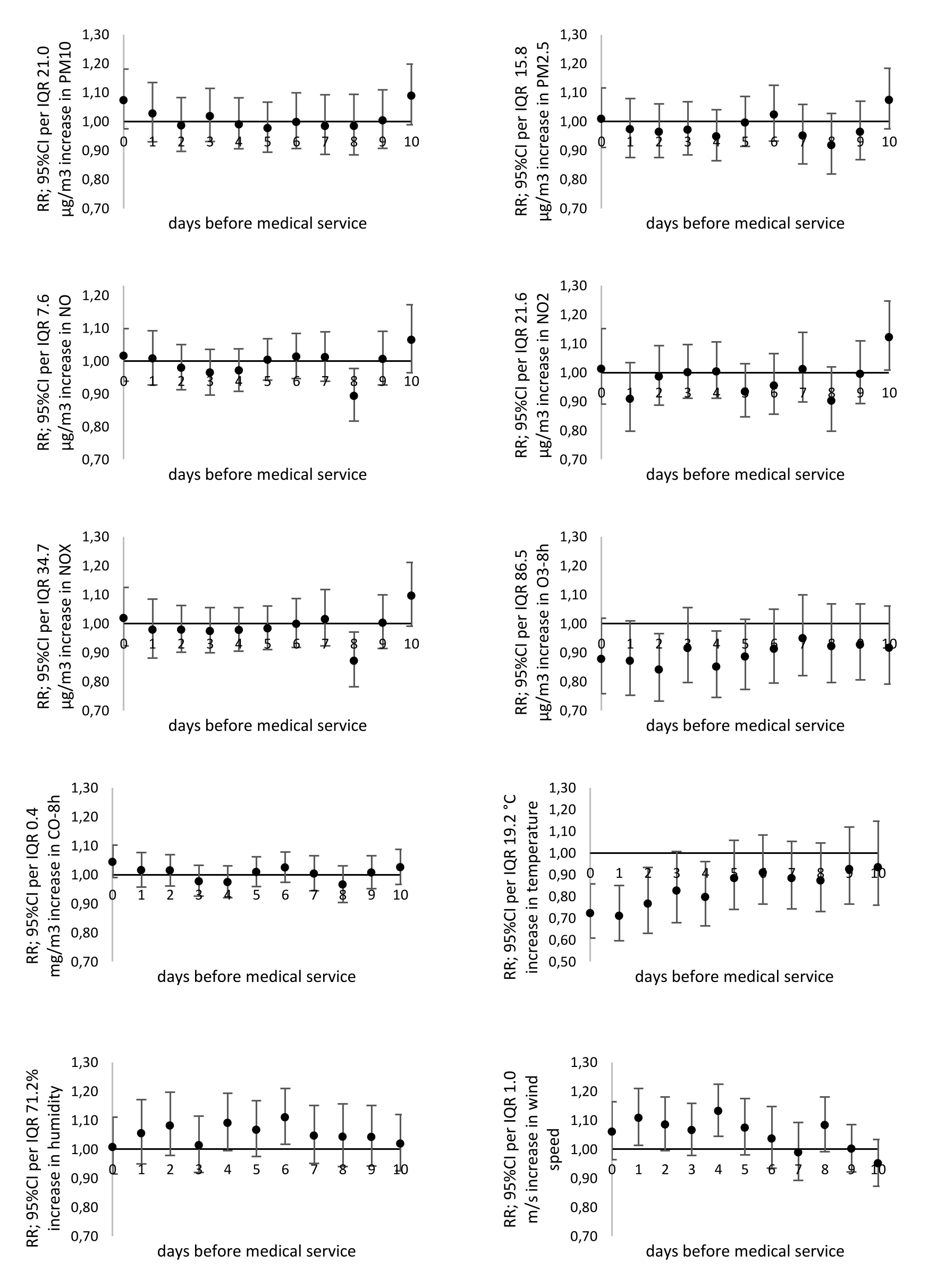

Supplement: S2 Fig — (TIF) [file pone.0335063.s005.tif]
